# Trends in the Incidence of Breast Cancer Following the Radiological Accident in Goiânia: A 25-Year Analysis

**DOI:** 10.31557/APJCP.2019.20.12.3811

**Published:** 2019

**Authors:** Rosemar Macedo Sousa Rahal, Marina Elias Rocha, Ruffo Freitas-Junior, Rosangela da Silveira Corrêa, Danielle Rodrigues, Edesio Martins, Leonardo Ribeiro Soares, Jose Carlos Oliveira

**Affiliations:** 1 *Advanced Center for Breast Diagnosis (CORA), School of Medicine, Federal University of Goias, *; 2 *Goiania Population-Based Cancer Registry, Goiania, Goias, Brazil. *

**Keywords:** Breast cancer, epidemiology, incidence, age group, Brazil

## Abstract

**Background:**

The largest radiological accident to occur in any urban area happened in Goiânia, Brazil, in 1987. One major concern regarding deleterious effects in the population was a possible increase in the breast cancer incidence. This study analyzed trends in the incidence of breast cancer over the 25-year period following the radiological accident.

**Methods:**

This ecological, population-based study was conducted to determine the incidence of breast cancer in female residents of Goiânia, Goiás, Brazil, between 1988 and 2012. The data were collected from the Goiânia population-based cancer registry. Crude and age-standardized incidence rates were calculated. The Joinpoint software program was used to calculate annual percent changes (APC) in the incidence of breast cancer.

**Results:**

Overall, 7,365 new cases of breast cancer were identified, with an annual crude incidence rate of 23.09/100,000 women in 1988 and of 71.65/100,000 women in 2012. The age-standardized incidence rate was 35.63/100,000 women in 1988 and 65.63/100,000 women in 2012. Analysis of the APC showed a significant annual increase of 4.8% in the incidence between 1988 and 2005 (p<0.0001) followed by stabilization in 2005-2012, with an APC of -3.5% (p=0.1).

**Conclusion:**

There was an increase in the incidence of breast cancer in the female residents of Goiânia, Goiás in the first 17 years of evaluation (1988-2004) followed by a period of stabilization until 2012. However, the trends in the incidence suggest a lack of association with the radiological accident.

## Introduction

The relevance of breast cancer worldwide is unequivocal, as corroborated by the International Agency for Research on Cancer (IARC) GLOBOCAN database, which shows the high incidence and mortality rates associated with this tumor compared to other types of cancer. Estimates for 2018 predicted 18 million new cases of cancer, with 11.6% of these referring to breast cancer (IARC, 2018). In addition, the mortality rate from breast cancer was expected to account for 6.6% of all deaths from cancer in 2018 (IARC, 2018).

In Brazil, the incidence of breast cancer has been gradually increasing over the years, with 59,700 new cases expected for 2019, corresponding to an estimated risk of 56.33 cases per 100,000 women (Brasil, 2019). It is a country of continental proportions and, according to the geographical region, different socioeconomic, cultural and racial profiles are observed, which could justify different incidence rates of the disease (Brasil, 2019). However, few studies have evaluated the temporal evolution of this incidence, mostly hospital-based studies. 

Certain risk factors have been described as being responsible for this increase, including delaying motherhood, changes in dietary habits, obesity, sedentariness, alcohol intake, increased life expectancy, the use of hormones and exposure to ionizing radiation (IAEA, 1988; Abellof et al., 2004). Nevertheless, most of these risk factors have low penetrance and, individually, controversy remains about the true impact as a causative factor for breast cancer.

The largest radiological accident to occur in any urban area happened in Goiania, Brazil, in 1987 (IAEA, 1988). The city is the capital of a Brazilian state located in the central region of the country, which had about 900,000 inhabitants at the time of the accident. This event was the consequence of the inappropriate removal and dismantlement of a sealed source from a medical radiotherapy device containing 50.9TBq of cesium-137. The accident caused widespread contamination of the central part of the city, resulting in four deaths and hundreds of people directly or indirectly affected by the accident (IAEA, 1988; Rosenthal et al., 1991). In total, the competent authorities monitored about 112,000 city residents, although their direct connection with the incident was not established (Rosenthal et al., 1991). About 15 years after the event, a case-control study analyzed the microsatellite mutation rates and found no difference between exposed and unexposed populations (Costa et al., 2011). Other studies have observed high cesium concentration in the subsoil layer of the accident site (Anjos et al., 2002) and indicated that exposure to ionizing radiation can be detected in offspring of exposed individuals (Cruz et al., 2008). 

One major concern regarding deleterious effects in the population was a possible increase in the rate of tumors, since there is evidence in the literature that exposure of an individual to ionizing radiation increases his/her risk of developing cancer, including breast cancer, as reported in studies conducted following the explosions of the Hiroshima and Nagasaki bombs (IAEA, 1988; McGregor et al., 1977). Therefore, the objective of the present study was to evaluate trends in the incidence of breast cancer over the 25-year period following the radiological accident.

## Materials and Methods

This was an observational, analytical, population-based study on the incidence of breast cancer conducted using data collected at the Goiânia population-based cancer registry (PBCR). The target population was women living in Goiânia, Goiás, Brazil who had been diagnosed with breast cancer between 1988 and 2012.

Data collected from the PBCR database included the patient’s name, date of birth and diagnosis. Whenever further data were required or when a variable required confirmation, a range of electronic databases were used, including the Goiânia PBCR, the Hospital Registry, and the Goiânia Municipal Health Department’s hospital database and hospital bed regulation system database. If any doubts persisted, further information was obtained from patients’ health records.

Based on the data collected, incidence rates were calculated: the crude rate, the crude rate by age group, and the age-standardized rate. To measure trends or changes in breast cancer rates over time, the annual percent change (APC) was calculated.

Calculation of the crude incidence rate was based on the ratio between the number of new breast cancer cases occurring between 1988 and 2012 and the number of individuals exposed to the risk of developing the disease (Boniol and Heaunue, 2009), as shown in equation 1.

*Equation 1:*

$\mathrm{Crude incidence rate}=\frac{\mathrm{number of new cases of cancer in the year}\times 100000}{\mathrm{Population of Goiânia in the year}}$

The crude incidence rate by age group was calculated in accordance with the ratio between the number of cases occurring in each age group and the size of the local population for each age group. This incidence rate was expressed per 100,000 inhabitants (Boyle and Parkin, 1991), as shown in equation 2.

*Equation 2:*

$\mathrm{Age}-\mathrm{standardized incidence rate}=\frac{\mathrm{Crude incidence rate by age group}\times \mathrm{standard population by age group}}{100000}$

Calculation of the age-standardized incidence rate was based on the world standard population as proposed by Segi (1960) and modified by Doll and Cook, (1967) and expressed per 100,000 inhabitants as shown in the following formula (Boyle and Parkin, 1991).

**Table 1:** The Frequency of New Cases of Female Breast Cancer According to Age Group in Goiânia, Goiás, Brazil Between 1988 and 2012

Age Group (years)	Total Number	Percentage
25-29	87	1.10%
30-34	321	4.30%
35-39	564	7.60%
40-44	921	12.50%
45-49	1,080	14.60%
50-54	1,047	14.20%
55-59	920	12.50%
60-64	717	9.70%
65-69	644	8.70%
70-74	436	5.90%
75-79	296	4.00%
≥ 80	289	3.90%

**Figure 1:**
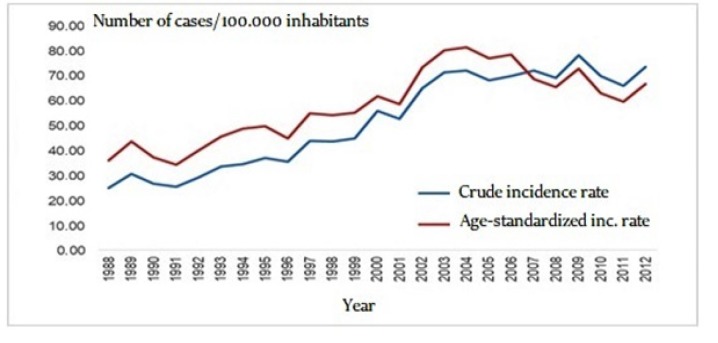
Crude and Standardized Incidence Rates of Female Breast Cancer in the City of Goiânia, Goiás, Brazil between 1988 and 2012

**Table 2:** Annual Percent Change in the Age-Standardized Incidence Rate of Female Breast Cancer in the City of Goiânia, Goiás, Brazil between 1988 and 2012 According to Age Group

Age Group (years)	Trend 1 (Years)	APC^*	(95%CI)	p-value	Trend 2 (Years)	APC^*	(95%CI)	p-value
25-29	1988-2008	4.6^	(1.6 – 7.7)	0.001	2008-2012	-19.5	(- 40.2 – 8.3)	0.1
30-34	1988-2012	3.3^	(1.7 – 5)	0.001	-	-	-	-
35-39	1988-2006	4.7^	(2.4 – 7.1)	0.001	2006-2012	-8.3	(- 16.3 – 0.5)	0.1
40-44	1988-2003	6.2^	(3.3 – 9.2)	0.001	2003-2012	-3.4	(- 7.1 – 0.4)	0.1
45-49	1988-2012	2^	(1 – 3.3)	0.0001	-	-	-	-
50-54	1988-2003	7.1^	(4.5 – 9.8)	0.0001	2003-2012	-4.6	(- 7.7 – 1.4)	0.001
55-59	1988-2003	7.1^	(4.2 – 10)	0.0001	2003-2012	-3.6	(- 7.1 – 0.1)	0.1
60-64	1988-2012	2.7^	(1.2 – 4.3)	0.0001	-	-	-	-
65-69	1988-2012	4^	(2.3 – 5.7)	0.001	-	-	-	-
70-74	1988-2012	0.5	(-0.7 – 1.7)	0.4	-	-	-	-
75-79	1988-2007	5.8^	(3.8 – 7.9)	0.001	2007-2012	-13.6	(- 21.5 – -5.0)	0.0001
≥ 80	1988-2012	0.9	(-0.9 – 2.8)	0.3	-	-	-	-

^Significance, p < 0.05; *, Annual Percent Change (APC); * Incidence rate per 100,000 women; CI, Confidence interval.

**Table 3.** Annual Percent Change in the Age-Standardized Incidence Rate of Female Breast Cancer by Age in the City of Goiânia, Goiás, Brazil between 1988 and 2012

Age Group (years)	Trend 1 (Years)	APC^*	(95%CI)	p-value	Trend 2 (Years)	APC ^*	(95%CI)	p-value
≤ 39	1988-2007	5.5	3.9 - 7.2	0.001	2007-2012	-7	-15.3 - 2	0.1
40 - 49	1988-2005	4.6	3.6 - 5.6	0.0001	2005-2012	-3.7	-5.7	0.0001
50 - 69	1988-2004	6.3	4.6 - 8	0.0001	2004-2012	-3.4	-6.2	0.0001
≥ 70	1988-2012	0.9	-0.1 – 1.9	0.1	--	-	-	-

^Significance, p < 0.05; *, Annual percent change (APC); *, Coefficient of the incidence per 100,000 women; CI, Confidence interval.

**Figure 2:**
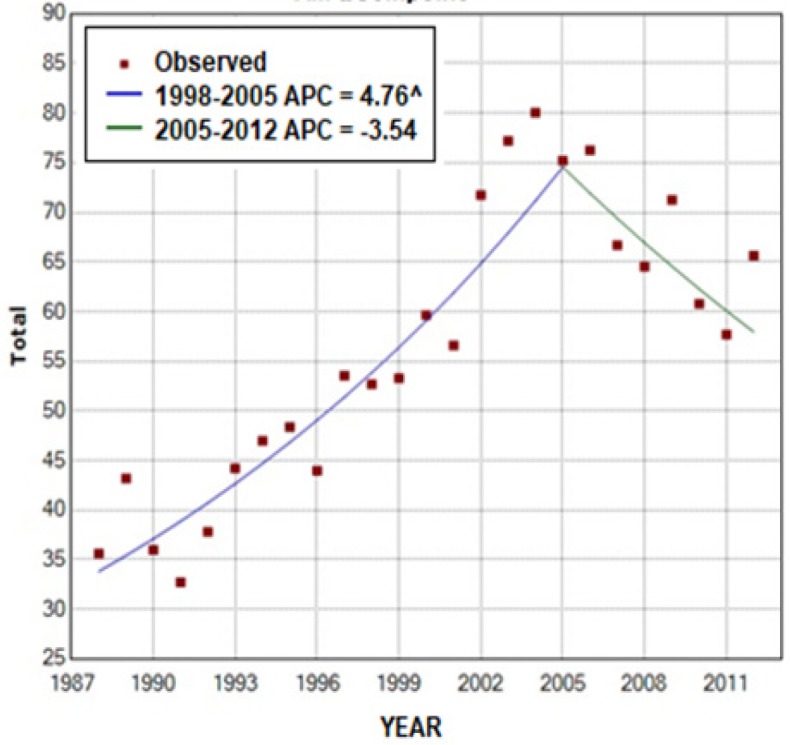
Analysis of the Annual Percent Change According to Women’s Age in the City of Goiânia, Goiás, Brazil between 1988 and 2012. ^Significance: p < 0.05

For the purposes of calculating both the incidence rates and the population at risk, age was stratified into 5-year intervals: 25-29, 30-34, 35-39, 40-44, 45-49, 50-54, 55-59, 60-64, 65-69, 70-74, 75-79 and ≥ 80 years.

The APC for the incidence trend of new cases of breast cancer was measured using the standard error calculation method adopted by Kim et al., (2000) and the Poisson regression using Joinpoint software, version 4.5.0.1 of June 2017 (Statistical Research and Applications Branch, Division of Cancer Control and Population Sciences, National Cancer Institute, USA) (NCI, 2018). Variables with p-values <0.05 were considered statistically significant and 95% confidence intervals (95%CI) were calculated. To interpret the results, the incidence of breast cancer was considered to have increased when there was an increase in the APC and the minimum value of the confidence interval was above zero. Conversely, the incidence was considered to have decreased when the APC decreased and the maximum value in the confidence interval was below zero. The incidence was considered to have stabilized when the minimum and maximum values in the confidence interval ranged from negative values to positive values.

*Ethical issues*

The study protocol was reviewed and approved by the internal review board of the Hospital Araújo Jorge, Goiás Anticancer Association, under number 1,629,547 and by the internal review board of the Federal University of Goiás Teaching Hospital under number 1,596,198. 

## Results

Between 1988 and 2012, 7,365 new cases of breast cancer were recorded in women. Analysis by age group showed that 13.2% of these new cases occurred in women aged ≤39 years, while 27.2% occurred in those of 40 to 49 years of age, 45.2% in women of 50 to 69 years of age and 13.8% in those ≥70 years of age. Table 1 shows the frequency of cases according to age group.

The annual crude incidence rate was 23.09 per 100,000 women in 1988 and 71.65 per 100,000 women in 2012. The age-standardized incidence rate was 35.63/100,000 in 1988 and 65.63/100,000 in 2012. There was an increase of 310% in the crude incidence rate and of 184% in the age-standardized incidence rate of breast cancer in women in Goiânia, as shown in Figure 1.

Analysis of the APC showed a statistically significant increase in the total number of cases of breast cancer of 4.8% per year between 1988 and 2005 (p<0.0001) followed by stabilization, as shown by an APC of -3.5% (p=0.01) between 2005 and 2012 (Figure 2).

When the incidence rate was stratified according to age group, consolidated into 5-year intervals, a significant increase was found throughout the entire evaluation period for the 30-34 year, 45-49 year, 60-64 year and 65-69 year age groups. For the 50-54 year and 75-79 year age groups, an initial increase in the APC (p=0.001) was followed by a decrease (p=0.001). For the 25-29 year, 35-39 year, 40-44 year and 55-59 year age groups, an initial increase (p=0.001) was followed by stabilization (p=0.1) (Table 2).

When age groups were grouped together, there was a significant increase in the APC between 1988 and 2007 followed by a decrease from 2007 to 2012 for those aged ≤ 39 years. For the 40-49-year and 50-69-year age groups, an initial increase in the APC (p=0.0001) was followed by a decrease (p=0.0001), while for the ≥70-year age group the APC was found to remain stable (p=0.1) (Table 3).

## Discussion

The results of this study suggest the occurrence of an increase in the incidence of breast cancer in the city of Goiânia, with an increase of 310% in the crude incidence rate and of 184% in the age-standardized incidence rate. This increase in the incidence is in agreement with the national and international literature (IARC, 2014; Ferlay et al., 2015; GBDCC, 2017). Nevertheless, this increase was not linear, since analysis of the 25-year period evaluated here shows a rise in the APC between 1988 and 2005 followed by a period of stabilization until 2012. 

Considering a latency time of 10 years for the development of breast cancer following exposure to radiation (Mørch et al., 2017), it was expected that there would be a peak in the incidence in 1997. However, the increase was gradual, and the incidence rate stabilized from 2005 onwards, suggesting a lack of association between the accident and the increased incidence. Other factors could justify this increase in the incidence of the disease, such as the improvement of diagnostic methods and the consolidation of mammographic screening in the city of Goiânia (Sadovsky et al., 2015; Freitas-Junior et al., 2016). 

When the analysis was stratified by age group, it was found that the frequency of breast cancer in young women, defined here as the 25-39 year age group, represented 13% of all the cases evaluated, with a significant increase in the APC, alerting to a need to investigate the possible factors associated with the development of breast cancer at this early age. This trend is in line with the rest of the country (Gravena et al., 2014) and cannot be justified by improved diagnostic methods, given that this population is not included in public screening policies (Migowski et al., 2018). Possible causes include the use of hormonal contraception and alcohol intake (McGregor et al., 1977; Seitz et al., 2012). Nevertheless, a population-based study conducted in Goiania found that this increase was similar to that observed in other age groups between 1988 and 2003 (Freitas-Junior et al., 2010). 

In relation to the use of hormonal contraception, there have been reports in the literature of a possible association between the use of these contraceptives and an increased risk of breast cancer. This subject is relevant bearing in mind that 140 million women of 15-49 years of age use this type of contraception at some time during their lives (WCP, 2013). There are reports in the international literature of an increase in the use of hormonal contraception, including a study conducted in Denmark where the percentage of women using hormonal contraception was found to have increased from 24% to 39% (Wilson et al., 2012; Lindh et al., 2017). A population-based study conducted in Brazil reported similar findings, with 75% of sexually active women of 10 to 19 years of age being in use of some contraceptive method and 61.8% of these women using oral contraceptives (Duarte et al., 2011; Olsen et al., 2018).

Mørch et al. added to the debate on a possible association between the use of hormonal contraceptives and the development of breast cancer when they reported an increased risk of breast cancer in users of hormonal contraceptives, with this risk remaining elevated even after the women had stopped use of the medication in relation to women who had never used hormonal contraception (Mørch et al., 2017, Olsen et al., 2018). 

Another factor that may also be playing a role in increasing the incidence of the disease in young women is alcohol abuse. This association has been widely investigated, particularly with respect to the type of alcohol consumed, the age at which alcohol consumption begins and the pattern of alcohol consumption (Scoccianti et al., 2014; Hanf and Gonder, 2005; Castro and Castro, 2014). A study conducted with Asian-American and Japanese-American women born in the United States showed that alcohol use was associated with a 15% increased risk of breast cancer even when consumption was limited to 5.0 to 9.9 grams/day or 3-6 doses/week (Wu et al., 2012). In Goiânia, there is a high prevalence of alcohol consumption among adolescents, as well as the early onset of alcohol use (Coutinho et al., 2016; Nascente et al., 2016). In addition, physical inactivity, inadequate eating habits and obesity may also have contributed to this increased incidence of the disease (Nascente et al., 2016; Godinho-Mota et al., 2018).

The data from the present study showed that one-fourth of the cases of breast cancer evaluated here occurred in women of 40 to 49 years of age. This is important information, since the Brazilian Ministry of Health recommendations for breast cancer screening do not include this age group (Migowski et al., 2018). In the absence of screening, these women are restricted to the diagnosis of advanced breast cancer, which favors more aggressive treatment and unfavorable clinical outcomes (Simon et al., 2019). 

For the 50 to 69 years age group, a significant increase was found in the APC up to 2004 followed by a significant reduction in the final eight years of the analysis. In fact, this decrease began in 2000 and coincides with the publication of the Women’s Health Initiative (WHI) study (Rossouw et al., 2002). In Brazil, as in other countries, a reduction occurred in the prescription of hormone replacement therapy (HRT) (Aquino et al., 2016), making it reasonable to assume that the reduction in the incidence of breast cancer found in this study could be related to the decrease in the use of HRT. 

The study’s limitation refers to the fact that secondary data were used; however, the data used in this study are from the Goiânia population-based cancer registry, which is recognized both nationally and internationally for its high quality, classified as A by the IARC (Moura et al., 2006).

In conclusion, there was an increase in the incidence of breast cancer in women living in Goiânia, Goiás, Brazil in the first seventeen years evaluated (1988-2004) followed by stabilization between 2004 and 2012. However, the trends in the incidence suggest a lack of association with the radiological accident.

## Abbreviations

APC: Annual Percent Change

HRT: Hormone Replacement Therapy

IARC: International Agency for Research on Cancer

PBCR: Goiânia population-based cancer registry
